# An Embodied Intelligence System for Coal Mine Safety Assessment Based on Multi-Level Large Language Models

**DOI:** 10.3390/s25020488

**Published:** 2025-01-16

**Authors:** Yi Sun, Faxiu Ji

**Affiliations:** School of Communication and Information Engineering, Xi’an University of Science and Technology, Xi’an 710054, China; 23307223003@stu.xust.edu.cn

**Keywords:** coal mine safety assessment, embodied intelligence, multi-source sensor data, multi-level architecture, logical reasoning

## Abstract

Artificial intelligence (AI), particularly through advanced large language model (LLM) technologies, is reshaping coal mine safety assessment methods with its powerful cognitive capabilities. Given the dynamic, multi-source, and heterogeneous characteristics of data in typical mining scenarios, traditional manual assessment methods are limited in their information processing capacity and cost-effectiveness. This study addresses these challenges by proposing an embodied intelligent system for mine safety assessment based on multi-level large language models (LLMs) for multi-source sensor data. The system employs a multi-layer architecture implemented through multiple LLMs, enabling not only rapid and effective processing of multi-source sensor data but also enhanced environmental perception through physical interactions. By leveraging the tool invocation and reasoning capabilities of LLM in conjunction with a coal mine safety knowledge base, the system achieves logical inference, anomalous data detection, and potential safety risk prediction. Furthermore, its memory functionality ensures the learning and utilization of historical experiences, providing a solid foundation for continuous assessment processes. This study established a comprehensive experimental framework integrating numerical simulation, scenario simulation, and real-world testing to evaluate the system through embodied intelligence. Experimental results demonstrate that the system effectively processes sensor data and exhibits rapid, efficient safety assessment capabilities during embodied interactions, offering an innovative solution for coal mine safety.

## 1. Introduction

Mine safety production directly affects miners’ lives and enterprise assets, making timely response and accurate assessment of mining alarm data particularly crucial [1]. These alarm data serve as the most direct and sensitive indicators of safety production, providing real-time reflections of operational conditions and potential safety hazards in mining operations. Therefore, rapid capture, precise analysis, and effective assessment of alarm data not only enable timely detection and resolution of safety risks to prevent accidents, but also significantly enhance the overall management level of mine safety production, thereby safeguarding people’s lives and property while maintaining social stability and harmony [2,3,4]. In essence, the assessment of alarm data constitutes an indispensable component of mine safety production, and its significance cannot be underestimated.

Based on the established limitations and prominent capabilities of LLM, embodied intelligence emphasizes the decisive impact of an intelligent agent’s physical existence and environmental interaction on intelligent behavior. This perspective diverges from traditional artificial intelligence’s reliance on abstract computational models, proposing a novel approach to achieving perception, learning, and action through dynamic interactions between body and environment. The concept of “embodied intelligence” heralds a critical transition toward truly autonomous systems [5]. LLMs are not merely linguistic tools but rather AI agents capable of interpreting, learning, and adapting to their environment. Through advancements in LLM, these capabilities have become increasingly feasible and have been demonstrated in recent studies, demonstrating exceptional performance across multiple domains ranging from design to decision making and path planning [6,7]. The application of embodied intelligence through multimodal LLMs has introduced new possibilities for autonomous mining operations, influencing the innovation and development of mining safety assessment systems [8].

Sensors provide favorable support for safety monitoring [9]. The acquisition and accurate assessment of sensor data are crucial. Traditional manual assessment processes typically require the involvement of trained experts who, upon receiving alarm data, begin a tense and detailed assessment process. This involves analyzing data trends, correlations, and historical sensor readings while referencing relevant national and industry standards to determine the nature of the alarm and necessary actions to be taken. However, due to the extensive range of monitoring targets and potential hazardous conditions at mine sites, this process demands considerable human, material, and temporal resources [10]. Moreover, the process relies heavily on the subjective judgment of assessment personnel, which can lead to subjective biases and potential oversight of critical issues [11]. Consequently, as the pursuit of optimal control at coal mine sites intensifies and challenges continue to escalate, this phenomenon increasingly emphasizes the urgent need for automated analysis and assessment.

In this paper, a novel approach is proposed: an embodied intelligent system for mine safety assessment based on multi-level LLMs. When alarm data are received, managers at the senior management level leverage their natural language understanding capabilities to analyze the scenario context of the received data, routing it to the corresponding scenario. Different scenarios then analyze the specific data type and route it to the assessment layer corresponding to that data classification. The assessment layer and the smallest assessment units begin to evaluate the data to determine whether it constitutes an alarm condition. If a single data classification cannot definitively confirm an alarm status, the system incorporates other data types and scenarios for comprehensive assessment.

The system integrates the cognitive and reasoning capabilities of LLM, performing precise reasoning and judgment through in-depth analysis of data and standards, combined with specific contextual information. The synergistic effect of its memory function and tool invocation further enhances the accuracy of LLMs during the judgment and decision-making process. The multi-level structure efficiently simulates the complex cognitive processes of humans, ensuring the system’s efficiency and precision in decision-making within complex scenarios. At the same time, the embodied intelligent system continuously interacts with the environment, constantly enhancing its adaptability and level of intelligence, thus maintaining exceptional performance in a variable environment. The system is capable of accurate judgment and decision making through the analysis of complex mine data.

The contributions of this paper are threefold: (a) implementing embodied intelligence for safety assessment through multi-level LLMs; (b) the employment of embodied intelligence to continuously interact with sensor data and automatically plan assessment pathways; and (c) the enhancement of assessment accuracy and reliability through human feedback integration.

The arrangement of the sections in this paper is as follows: Section 1 introduces the background and shortcomings of embodied intelligence and artificial judgment; Section 2 provides a brief review of the literature related to this study; Section 3 describes the system framework designed in this paper, as well as the data sources and key nodes; Section 4 presents the experimental validation; Section 5 evaluates the experimental results; Section 6 discusses the experiments and their findings; Section 7 concludes the study; and Section 8 outlines directions for future research.

## 2. Related Work

In this section, we review the existing literature related to our study. We first explore the current state and capabilities of embodied intelligence. Subsequently, we discuss the methods and technologies currently used for coal mine judgment and decision-making, highlighting their limitations and the need for more advanced approaches. Then, we examine the latest advancements in LLM and their applications across various fields. Finally, we investigate the impact of hierarchical structures on LLMs. These discussions provide a foundation for the framework we propose.

### 2.1. The Current State of Embodied Intelligence Research

Embodied intelligence, grounded in artificial intelligence, is driving the evolution of future intelligent systems, with its core focus on endowing robots with the capability for multimodal physical interaction and continuous evolution in open-world environments. Research demonstrates that embodied intelligence exhibits significant potential across various domains, particularly when enhanced by foundational AI models, enabling more sophisticated and adaptive production processes [12,13]. In this context, the development of embodied neural intelligence is particularly noteworthy, as it focuses on applying neuromorphic technologies to robotics, achieving tight integration from perception to motor control [14]. Related studies emphasize the promise of this approach in creating environmentally integrated robotic systems, providing new perspectives for the future advancement of robotics technology.

Research on embodied intelligence extends beyond the technical realm, delving into how humans perceive the world around them and integrate this information for planning and decision making. By simulating human perceptual and cognitive processes, embodied intelligence provides robots with more humanized interaction capabilities, enabling them to perform more flexibly and intelligently in complex, dynamic environments. Research incorporates semantic context and state transitions of previously interacted objects into action sequences to infer subsequent behaviors, significantly enhancing their capabilities even in novel environments [15]. Research drawing upon the concept of expert conferences, demonstrates that effective communication and deliberation facilitate the perception of instruction-relevant information, correct unexpected errors, and filter out inconsistent action decisions [16]. Research generates executable plans based on existing objects in the scene through the alignment of LLM with visual perception models [17].

In the application of embodied intelligence, the capacity for multimodal physical interaction enables robots to execute increasingly complex tasks in real-world environments. For instance, service robots equipped with visual, auditory, and tactile sensors can accurately interpret human needs and deliver precise services across domains such as healthcare, household, and retail settings. Embodied intelligence has found practical applications in the education sector, manufacturing industry and healthcare field [18,19].

Furthermore, the continuous evolution capability of embodied intelligence implies that robots can learn from experience and continuously optimize their behavioral patterns, allowing them to make more informed decisions when faced with unfamiliar situations.

The development of embodied intelligence has also advanced human–robot collaboration. On industrial production lines, robots equipped with embodied intelligence can work alongside human workers, enhancing productivity and safety by understanding human actions and intentions. In the research domain, simulations and experiments involving embodied intelligence offer new tools and methods for fields such as cognitive science, psychology, and neuroscience, aiding in the exploration of the nature of human intelligence.

### 2.2. The Research Status of Intelligent Judgment and Analysis

Coal mine safety monitoring plays a crucial role in the safety construction and normal operation of mines. Effective safety monitoring can provide real-time insights into the environmental conditions within the mine, promptly detect and eliminate safety hazards, and prevent major accidents such as gas explosions, water inrushes, and collapses. This necessitates timely and accurate assessment of alarm data; traditional manual assessment is limited by human resources and is inefficient [20,21]. To overcome these limitations, the development of intelligence and informatization is an inevitable trend for the safety production of coal enterprises. The latest research is dedicated to utilizing automation technology and intelligent systems to enhance safety monitoring. For instance, some researchers have investigated the application of unmanned aerial vehicles and Internet of Things (IoT) technology in tunnel and mine monitoring, demonstrating how they can serve as auxiliaries to intelligent monitoring systems and improve decision-making efficiency [22,23]. Additionally, research has focused on the application of big data analysis in intelligent risk assessment [24,25]. Some studies focus on the safety assessment of coal mine gas explosions, establishing safety risk assessment models to reduce risks [26,27]. These studies indicate that, through the integration of big data analysis and sensor data, the scope of coal mine safety monitoring can be effectively expanded, and the risks faced by personnel during risk assessment can be reduced.

With the development of data-driven and intelligent technologies, the application of methods such as artificial intelligence and machine learning has brought new opportunities for the optimization of intelligent monitoring systems [28,29,30]. The use of advanced algorithms and predictive models to automate the monitoring and alarm process has improved the accuracy and efficiency of safety monitoring. Retrieval-Augmented Generation (RAG) and standard-based reasoning are also important components of intelligent assessment systems. The knowledge base enhances the diversity of retrieved texts, providing strong support for the assessment of alarm data [31]. AI technologies and data-driven models have increased the efficiency of risk assessment and ensured worker safety and project compliance [32,33,34]. The application of these technologies not only enhances the efficiency of assessment, but also helps to ensure the safety of coal mine workers.

Additionally, the forward direction of intelligent monitoring system technology indicates that enhancing sensor networks and artificial intelligence analysis techniques will play a key role in the field of gas monitoring and safety management in the future. Intelligent predictive analysis highlights its potential to improve the efficiency of safety monitoring [35]. The intelligent upgrade of coal mine safety monitoring not only enhances the management level of mine safety production, but also provides a more solid guarantee for the life safety of miners. The integration of artificial intelligence and machine learning technologies allows safety monitoring systems to more accurately predict and identify potential safety risks, enabling preventive measures to be taken before accidents occur. Furthermore, through data-driven decision support systems, coal mining enterprises can more effectively utilize monitoring data, optimize resource allocation, and improve emergency response capabilities. In the future, with the continuous advancement and innovation of technology, there is reason to believe that intelligent coal mine safety monitoring systems will become a key force in promoting the standardization and modernization of safety production in the coal industry, making an even greater contribution to the sustainable development of China’s coal industry.

### 2.3. Current Research on the Reasoning Ability and Hierarchical Structure of LLM

In recent years, LLMs have made remarkable progress. The latest research indicates that LLMs have demonstrated unprecedented potential in a wide range of application scenarios, becoming a key driving force in the development of intelligence. For instance, GPT-4 has achieved higher accuracy in text generation and context understanding, thereby expanding the application domain of pre-trained models [36].

With the significant improvements in the natural language understanding and reasoning capabilities of LLM, researchers have begun to explore how to more effectively harness the potential of these models to tackle more complex tasks. In order to further leverage their reasoning abilities, methods such as step-by-step thinking and chain-of-thought have been developed [37,38]. Against this backdrop, a tree-like thinking structure inspired by the human problem-solving approach—known as “Thinking Trees” (ToT)—has been introduced into the reasoning framework of LLMs to further enhance the models’ capability and efficiency in solving complex problems [39]. ToT expands on the popular “chain-of-thought” approach and allows for the exploration of coherent text units (“thoughts”) as intermediate steps in problem-solving. By adding a memory module that records the dialogue and state history of the problem-solving process, the system can backtrack to earlier steps in the thought process and explore other directions from there [40]. LLMs utilize a broader perspective for querying and reasoning, thereby enhancing their capabilities. With a hierarchical structure, LLMs have a wider field of view, capable of global reasoning by leveraging information from sub-nodes, thus recovering from local errors [41].

LangChain and LangGraph have enhanced the reasoning capabilities of models when dealing with complex tasks by integrating LLM with other data sources through chain-like or graph structures, accelerating the application of LLM in AI application development and demonstrating their potential in promoting the rapid development of LLM [42,43]. With the continuous advancement of large models, their natural language understanding and reasoning abilities are being applied in many industries, including legal text analysis, medical diagnosis and financial risk assessment, among others [44,45,46]. These applications fully reflect the deep integration of LLM in specialized fields and their broad application potential.

Although LLMs and hierarchical structures have made significant progress in various fields, research and practical applications in specific areas such as coal mine safety monitoring are still very limited. Currently, coal mine safety monitoring and assessment primarily rely on manual execution and analysis, which is not only time-consuming and labor-intensive but also has limitations in real-time data processing in high-risk or complex environments. Furthermore, although there is research on using Internet of Things (IoT) technology to assist with safety monitoring, these technologies typically do not include the advanced use of LLM, hierarchical structures, and RAG techniques for understanding and assessing alarm data.

To bridge this gap, the research presented in this paper aims to enhance the efficiency and accuracy of assessment by interacting with real-time sensor data through a multi-level structured LLMs, performing step-by-step and hierarchical assessment processing to determine whether the data constitute an alarm. This approach is expected to improve the efficiency and accuracy of assessments, particularly when dealing with complex tasks and data analysis.

## 3. Methodology

In this section, the designed system is described in detail, including its various components and hierarchical structure. The main focus is on automatic path planning and design, standardization and data matching, intelligent assessment, human feedback, and preliminary implementation of embodied intelligence. This part encompasses the key aspects of system design, enabling accurate judgment and decision making for the complex data associated with mines.

### 3.1. Automatic Path Planning and Design

**Figure 1:**
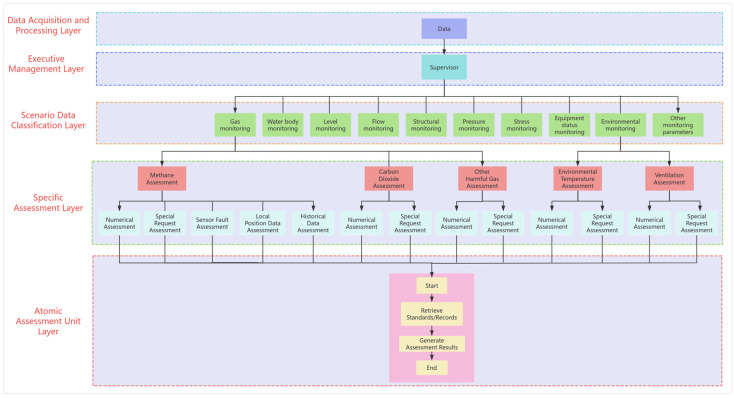
Multi-level hierarchical structure diagram.

This paper adopts a hierarchical management design concept to achieve multiple objectives within the system, including clear division of labor, streamlined processing, improved processing efficiency, ease of management and maintenance, flexibility and scalability, specialized judgment, reduced error rates, enhanced system stability, promoted information flow, and establishment of a closed-loop feedback mechanism. This management structure ensures the system’s stable operation and accurate judgment in complex mining environments. Through progressive data-processing and decision-making procedures, the overall system performance and work efficiency are effectively enhanced. The entire system is divided into five layers: the data collection and processing layer, the senior management layer, the scene data classification layer, the specific judgment layer, and the minimum judgment unit layer, as shown in Figure 1.

Within the constructed hierarchical structure, the leading LLM has demonstrated exceptional decision-making capabilities. It can intelligently plan the most suitable judgment paths based on real-time data information. This ability makes the LLM particularly efficient in handling complex issues. At each individual level, the LLM exhibits an adaptive path selection mechanism that not only quickly analyzes the specific needs of the current level but also dynamically adjusts the decision-making direction in response to data changes. The combination of this flexibility and adaptability ensures the smoothness and efficiency of the entire decision-making process, thereby significantly enhancing the rate of problem-solving while maintaining decision quality. Whether in data filtering, information integration, or the final decision-making stages, the LLM can intelligently navigate to ensure that each step is an optimal choice, thereby optimizing the effectiveness of the entire decision-making process.

Data Acquisition and Processing Layer: This layer executes precise and efficient acquisition of monitoring data from diverse sensor arrays. These sensing devices may encompass multiple types of monitoring equipment for detecting roof displacement, gas concentration, dust content, hydrological conditions, and equipment operational status. The acquired data typically exist in analog or digital signal formats, necessitating a sequence of preprocessing operations before utilization, including data cleansing, validation, completion, desensitization, and distillation. These preprocessing procedures are crucial for enhancing data usability and ensuring the accuracy of subsequent assessments. Through these processing operations, the system can identify and eliminate erroneous data, thereby ensuring that the information transmitted to the assessment system maintains reliability and validity.

Executive Management Layer: The senior management layer, as the top-level design of the entire system, plays a crucial role in command and decision making. At this level, there is only one supervisor, whose role is akin to the “brain” of the system. The responsibilities of this supervisor are of paramount importance. The supervisor possesses highly developed natural language understanding capabilities, deeply parsing natural language inputs from users or data from sensors. The supervisor can understand the linguistic expression of data much like a human, ensuring an accurate grasp of the data’s content. Whether this consists of environmental data from sensors or other data input by users, the supervisor can quickly identify the specific context of the data and route it to the next-level scene data classification layer.

Scene Data Classification Layer: This layer is primarily composed of supervisors for 10 monitoring scenarios. They categorize the data received from the senior management layer’s Supervisor into specific categories. For instance, the Gas Monitoring Supervisor for the gas monitoring scenario would classify the data received from the senior management layer’s Supervisor into categories such as methane, carbon dioxide, and other harmful gases; the Environment Monitoring Supervisor for the environmental monitoring scenario would categorize the data into ventilation, ambient temperature, etc. Additionally, this layer is responsible for the flow of assessments to the next level, where data are routed for assessment based on the received data and predefined logical criteria.

Specific Assessment Layer and Atomic Assessment Unit Layer: The specific judgment layer, which is the bottommost but crucial level of the entire system, is where the actual assessments take place. It consists of a series of specialized judgment nodes, forming a tight network of assessments. Based on the received data, it searches for corresponding standards in a vector database, then compares the standards with the data to determine whether they constitute an alarm.

Through this multi-level and multi-perspective assessment, the specific judgment node layer ensures the comprehensiveness and effectiveness of mine safety monitoring, providing solid technical support for the safe production of mines. Additionally, the hierarchical structure offers a clear approach for path planning, which is beneficial for the design and planning of assessment paths by LLM.

### 3.2. Standardization and Data Matching

The minimum judgment unit conducts specific assessments based on the data it routes. The process primarily consists of two stages. The first stage is the data retrieval and comparison phase, where the sensor data are searched against a knowledge base to find the corresponding standard documents. These standard documents contain pre-set thresholds, ranges, patterns, or any other benchmarks used to determine the normalcy of the data. The second stage is the judgment phase, where, upon successfully retrieving the relevant standard documents, the minimum judgment unit begins an in-depth analysis of the sensor data, evaluating whether they exceed the pre-set thresholds or match any abnormal patterns. If the data points fall within the normal range, the system deems them to be in a normal state; conversely, if the data points are outside the range or match abnormal patterns, the system flags them as alarm data.

#### 3.2.1. Data Sources

In the process of mining production, safety is a crucial indicator. To ensure mine safety, a series of advanced sensor systems are typically deployed in the mining environment. These sensors act as the “nerve endings” of the mine, capable of accurately and real-time monitoring various key indicators within the mine. Specifically, they can capture physical and chemical quantities such as temperature changes, humidity levels, barometric conditions, and concentrations of harmful gases, thereby providing strong data support for the safe production of the mine.

**Figure 2:**
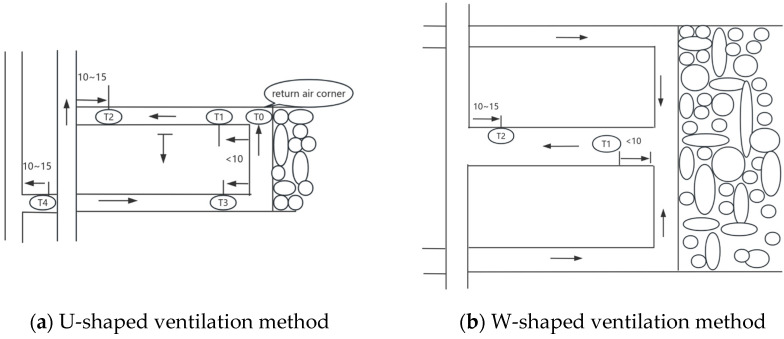
The longwall coal mining face sensors are arranged according to this figure. In the U-shaped ventilation method, a methane sensor T0 is set at the return air corner (less than 1 m from the cut roof line); a methane sensor T1 is set at the working face; a methane sensor T2 is set in the return airway of the working face; in the coal and gas outburst mine, methane sensors T3 and T4 are set in the intake airway. When using series ventilation, a methane sensor T4 is set in the intake airway of the worked-on face, as shown in (**a**). The arrangement of methane sensors for the W-shaped ventilation form of the coal mining face follows the aforementioned regulations, as depicted in (**b**). (The units in this figure are in meters).

As illustrated in the schematic diagram of sensor installation (Figure 2), we can see that these sensors are deployed at various key locations within the mine. In the depths of the mine, temperature sensors can detect heat changes generated by geological activities or the operation of mechanical equipment, ensuring that the temperature within the mine remains within a suitable range; humidity sensors provide real-time feedback on the humidity conditions inside the mine, preventing equipment malfunctions or mine collapse accidents due to excessive humidity; pressure sensors play a crucial role in monitoring changes in internal air pressure within the mine, promptly detecting abnormal situations, and preventing dangers caused by sudden pressure changes.

After acquiring raw data through sensors, to ensure the accuracy and effectiveness of subsequent assessment processes, we first need to perform a series of preprocessing steps on the data. Firstly, we ensure that the data obtained from different sensors are aligned in terms of timestamps to facilitate correct correlation of data from various sensors during subsequent analysis. The data from multiple sensors are integrated according to a unified reference coordinate system to enable comprehensive analysis. Duplicate records in the data are removed to avoid introducing bias during analysis. All data are converted into a unified format, such as time-series data or structured tables.

Through the aforementioned preprocessing steps, we are able to provide a clean, unified, and high-quality dataset for the assessment system, which lays a solid foundation for subsequent analysis. The preprocessed data will be more suitable for in-depth statistical analysis, pattern recognition, and the construction of predictive models, thereby enhancing the accuracy and reliability of the assessment system.

#### 3.2.2. Knowledge Base Enhanced Retrieval

The reasoning capabilities and content of large models often rely on the data they have been trained on. In today’s rapidly evolving information environment, the reasoning abilities of these models need to continually adapt to new data and knowledge. This is where the knowledge base becomes particularly crucial. A knowledge base is a system that integrates a vast amount of information resources. By organizing, categorizing, and indexing knowledge, it enables quick and convenient retrieval and utilization of this knowledge.

Knowledge bases come in various forms, and vector databases, as one type of knowledge base, are capable of abstract representations of diverse data types such as text, images, and audio. By calculating the similarity between vectors, vector databases can achieve rapid and accurate approximate retrieval. As a specialized data management tool, vector databases provide an ideal solution for the needs of large models. By mapping objects to vectors in high-dimensional space, vector databases not only facilitate real-time data updates but also ensure that large models can quickly and accurately locate the most relevant information when dealing with complex issues. Vector databases are specifically designed for storing and retrieving multi-dimensional vector data. They store data through vector embedding techniques, mapping various objects to vectors in multi-dimensional space. Each object corresponds to a vector that captures the object’s diverse features or attributes. Similar objects are close to each other in vector space, while dissimilar objects are farther apart. Depending on the complexity and detail of the data, the dimensionality of each vector can range from a few to several thousand. This multi-dimensional vector representation method allows for rapid and accurate data localization and retrieval based on the vector proximity or similarity of the data. Vector databases use specific similarity measures to find the closest matches, employing “approximate nearest neighbor” search techniques, including methods such as hashing and graph-based searches.

This paper integrates relational and vector databases to form a comprehensive knowledge base. Initially, using advanced word embedding techniques, key textual information such as standards, regulations, and safety protocols followed during assessments are successfully transformed into vector representations in high-dimensional space. These vectors not only encapsulate the semantic information of the original text but also mathematically express the similarity and correlation between texts. These vectorized pieces of knowledge are stored in a specialized vector database, creating an efficient knowledge storage and retrieval system. A large amount of alarm data, after being evaluated, is stored in a relational database to provide historical data support for subsequent assessments.

Building upon the knowledge base, we implemented an enhanced retrieval technology based on RAG. The application of this technology significantly improves the precision and applicability of assessment criteria. Utilizing RAG, the system can not only swiftly retrieve historical cases and assessment criteria closely related to the current judgment task from the knowledge base, but also integrate and distill this information through an intelligent generation process. This provides a more comprehensive and in-depth reference for judgments.

In conjunction with the vector database utilized in this paper, RAG primarily relies on the Hierarchical Navigable Small World (HNSW) algorithm within approximate nearest-neighbor algorithms, as well as the vector similarity search algorithm built into Chroma. The HNSW algorithm constructs a multi-layer graph structure where each data object has a corresponding node in the bottom layer, with these nodes being closely connected. As the number of layers increases, the number of data points in each layer decreases exponentially, yet they remain a subset of the points in the lower layers, maintaining a certain degree of structural similarity. During the search process, the algorithm begins at the highest layer to locate the nearest data points and progressively refines the search by descending layer by layer until the precise nearest neighbors are found at the lowest layer. This hierarchical structure enhances search efficiency while maintaining high search accuracy.

Under this operational paradigm, the system continuously learns and evolves through each assessment iteration, optimizing its evaluation logic and rendering the entire assessment process more intelligent and precise. This continuous self-optimization and iteration significantly enhances both the efficiency and quality of the assessment work, ensuring the reliability and scientific validity of assessment outcomes. Through these technological approaches, the system not only achieves comprehensive utilization of historical experience, but also advances assessment practices toward greater efficiency and intelligence.

### 3.3. Intelligent Assessment

Upon receiving data, the intelligent assessment module leverages the natural language-understanding capabilities of LLMs to route data to corresponding scenarios for specific assessments. Based on functionality, purpose, and risk management considerations, this paper categorizes monitoring scenarios into the following types: gas monitoring, environmental monitoring, structural monitoring, water body monitoring, pressure monitoring, level monitoring, equipment status monitoring, stress monitoring, flow monitoring, and other monitoring applications. These classified data facilitate the identification and monitoring of key safety indicators, enabling timely warnings and responses to potential hazards, thereby enhancing safety. Furthermore, appropriate management measures are implemented for different categories; data analysis and trend forecasting are conducted to improve decision-making accuracy; equipment status monitoring enables timely maintenance and repairs, reducing failure rates; and compliance with mine safety regulations and standards is ensured. A detailed classification of scenarios is presented in Table 1.

**Figure 3:**
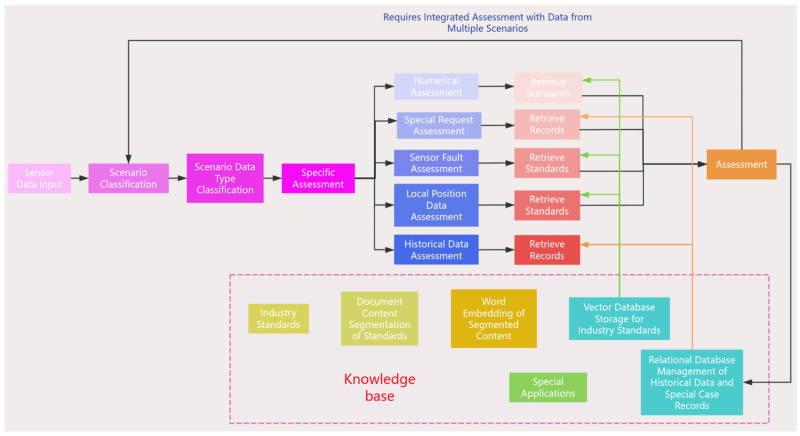
Analysis and alarm process flowchart.

The intelligent assessment and alarm module receives real-time data, including methane, carbon dioxide, other harmful gases, environmental temperature, wind speed, etc. The framework of the assessment process is shown in Figure 3.

The intelligent assessment module is a core component of the intelligent assessment system, which rigorously adheres to national safety standards and industry specifications for various assessment tasks. The intelligent assessment module integrates advanced algorithms such as machine learning, deep learning, and pattern recognition, as well as intelligent analysis techniques like data mining and knowledge graph. The integration of these technologies enables the module to efficiently process and analyze the complex environmental conditions and dynamically changing data in mines.

For the assessment of gas monitoring scenario data, the first step is to verify whether the values of sensors with specific locations and numbers exceed the limits according to standard documents. If values are within limits, the data are recorded; if limits are exceeded, the process advances to special application assessment. If a corresponding special application exists, indicating non-alarm data, the data are recorded and the assessment process concludes; if no corresponding special application exists, the process proceeds to sensor malfunction assessment. In cases of sensor malfunction, indicating non-alarm data, the data are recorded and measures are proposed according to standards, concluding the assessment process; if no sensor malfunction is detected, the process advances to local data assessment. When local data exhibit abnormality, a local data anomaly alarm is triggered, concluding the assessment process; if local data are normal, the process proceeds to historical local data assessment. If comparison with historical data from the measurement point indicates current data abnormality, a local data anomaly alarm is triggered, concluding the assessment process; if local data are determined to be normal, the assessment proceeds to other scenarios, specifically environmental scenarios. Within the environmental scenario, wind speed assessment is conducted initially. If gas data abnormality is attributed to abnormal wind speed, an alarm is triggered; if wind speed is normal, environmental temperature assessment follows. If environmental temperature is abnormal, an alarm is triggered; if environmental temperature is normal, the case is forwarded for in-depth manual assessment.

For the assessment of environmental monitoring scenario data, the first step is to verify whether the values of sensors with specific locations and numbers exceed the limits according to standard documents. If no limits are exceeded, the data are recorded; if limits are exceeded, the process advances to special application assessment. If there is a corresponding special application, it indicates that the data are not an alarm, and the data are recorded, ending the assessment process; if there is no corresponding special application, the next step is to assess sensor malfunction. If there is a sensor fault, it indicates that the data are not an alarm, the data are recorded, and measures are proposed according to standards, ending the assessment process; if there is no sensor fault, an alarm is initiated.

### 3.4. Human Feedback

Throughout the assessment process, each node interacts closely with human operators, forming a dynamic feedback loop mechanism. This mechanism ensures the flexibility and adaptability of the system, allowing it to be adjusted according to actual conditions. When an assessment is conducted at each node, the system displays the corresponding standard document search results and assessment outcomes in real-time to the monitoring personnel. The staff, relying on their professional knowledge and experience, review and evaluate the assessment results provided by the system.

When the personnel acknowledge the assessment results by providing positive feedback, the system deems the current assessment process and outcomes to be in line with the actual situation. In this case, the system will continue to proceed to the next assessment node or execute the corresponding operational commands as predefined. Positive feedback not only confirms the system’s performance but also serves as a crucial basis for the system’s continuous learning and optimization. If, during the assessment process, the personnel identify deviations or inaccuracies in the assessment results, i.e., negative feedback, the system will respond immediately. Upon receiving negative feedback, the system will suspend the current workflow. The negative feedback mechanism is a key component of the system’s self-correction and continuous improvement. Whether positive or negative, the system meticulously documents all feedback, which will be used for subsequent system optimization and model training. By continuously learning from the feedback of human operators, the system can progressively enhance the accuracy and efficiency of its assessments.

### 3.5. Preliminary Implementation of Embodied Intelligence

**Figure 4:**
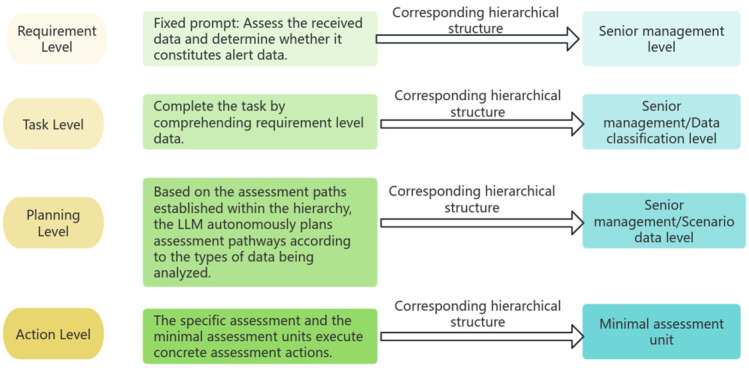
Embodied intelligence analysis diagram.

Embodied intelligence is divided into four levels: the requirement level, the task level, the planning level, and the action level. These four levels constitute the complete process of an intelligent agent, from perceiving environmental demands to executing specific actions. The demand level is responsible for understanding the agent’s goals and motivations within the environment, translating intrinsic and extrinsic needs into concrete task objectives. Subsequently, the task level refines these needs into a series of actionable tasks, providing clear direction for the agent’s actions. Following this, the planning level formulates the steps and strategies to accomplish the tasks based on the current environment and the agent’s state, ensuring the agent can complete tasks efficiently. Finally, the action level carries out these planned actions, influencing the environment through physical interaction, and in this process, fulfilling the agent’s goals and needs. The entire process reflects the characteristics of embodied intelligence of the agent, transitioning from abstract to concrete, from planning to execution. Figure 4 illustrates the corresponding relationship between the levels.

**Figure 5:**
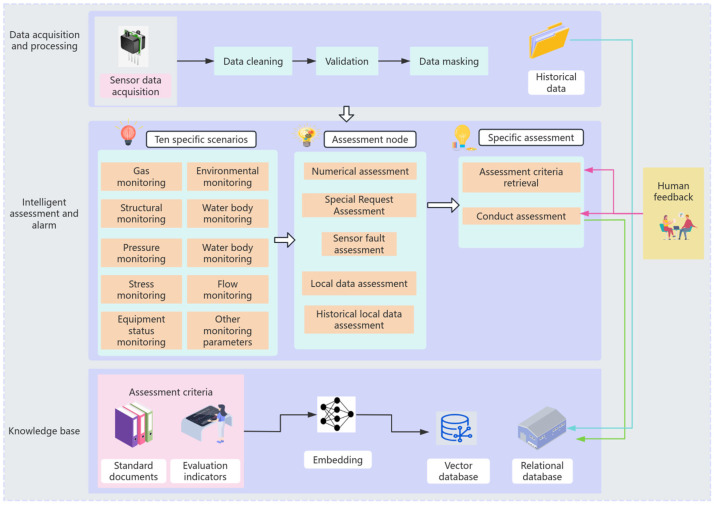
Intelligent decision-making and alarm system architecture.

The embodied intelligence system for assessment constructed in this paper, as shown in Figure 5, is a complex and powerful process designed to achieve comprehensive analysis and assessment of mine data. The system begins with the reception of data, starting with data collection and processing. Real-time data are sourced from sensors and monitoring equipment, etc. To ensure data quality and consistency, the received data must undergo cleaning and preprocessing, such as removing noise, filling in missing values, standardizing formats, etc. The cleaned data are then sent to the management layer, where the scenario of the data is identified, and the data are further directed to the corresponding scenario. The data classification layer sorts the data into specific categories and sends it to the specific assessment layer to begin the assessment process.

## 4. Experiments

In this paper, we will utilize the powerful development platform VSCode 1.96, along with the Python 3.11.10 programming language, to comprehensively validate the proposed system. The entire experimental process, including data processing and storage, is efficiently completed within a local environment. In terms of data storage, we employ two types of databases: one is the vector database Chroma, renowned for its efficient data retrieval performance; the other is the widely used relational database sqlite3, which is favored for its stability and ease of use in numerous projects. Furthermore, the hierarchical structure is based on langGraph 0.2.39, with the LLM model being the state-of-the-art GPT-4o, and the word-embedding model utilizing the Embedding technology provided by OpenAI 1.52.0. The integration of these two models provides strong support for the validation of our system.

### 4.1. Automatic Path Planning and Design

In the study of route planning, data from five categories in two detection scenarios were selected, with 20 groups of data for each category to conduct experiments. The success rate was defined as the ratio of successfully planned routes to the total number of routes. The selected scenarios were gas monitoring and environmental monitoring. The categories selected for the gas monitoring scenario included methane, carbon monoxide, carbon dioxide, and other harmful gases, while for the environmental monitoring scenario, environmental temperature and ventilation were considered. The path planning process began with the high-level Supervisor and consisted of five planning stages: The first planning stage determined which scenario to route to; the second stage planned the routing to specific data types within the scenario; the third stage planned the routing to specific assessment nodes; the fourth stage determined whether additional assessment nodes within the same classification were required; and the fifth stage evaluated whether data from other monitoring scenarios should be incorporated for joint assessment.

**Figure 6:**
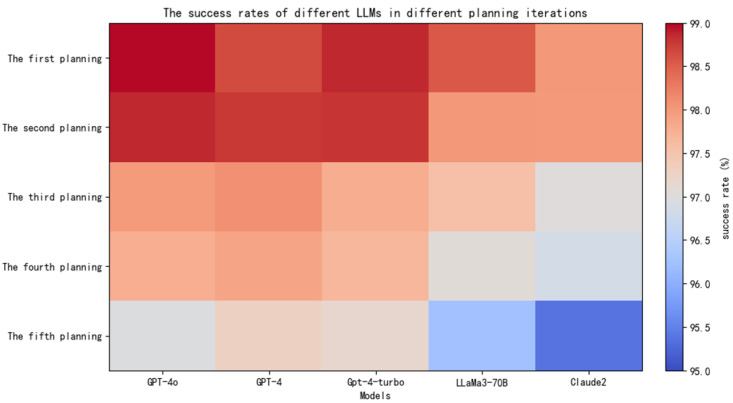
Heatmap of path planning under different LLMs.

Figure 6 presents a heat map of path planning, where the color gradient indicates the success rate levels, with dark red representing high success rates and light blue indicating lower success rates. The color bar on the right shows the specific percentages ranging from 95% to 99%. As illustrated in the figure, the first path planning achieved the highest success rate, at 98.97%. Although the success rate of the second planning stage showed a slight decrease, it maintained a satisfactory level. The third planning stage demonstrated generally good performance. However, starting from the fourth planning stage, the success rates were notably lower, all falling below 96.5%. As the hierarchical structure deepened, the tasks became increasingly complex, leading to deviations in the system’s path-planning capabilities.

### 4.2. Standardization and Data Matching

To compare the performances of different LLMs in the retrieval and comparison phase, as well as in the assessment phase, we conducted 20 experiments with the same data and prompts. After the first phase, the success rate of the retrieval and comparison phase was evaluated by calculating the ratio of the correct number of retrieved documents to the total sample size. Following the completion of the second phase, the success rate of the assessment phase was evaluated by the ratio of the correct number of assessment results to the total sample size.

**Figure 7:**
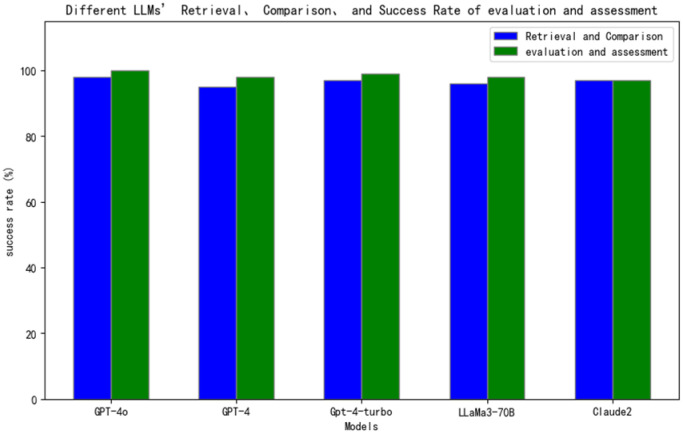
Matching of standards with data under different LLMs.

Figure 7 demonstrates that various models exhibited excellent performance in the retrieval and comparison phase. We conducted a comparative analysis of five models—GPT-4o, GPT-4, GPT-4-turbo, LLaMa3-70B, and Claude 2—evaluating their success rates in retrieval, comparison, and assessment tasks using identical datasets. Notably, GPT-4o demonstrated superior performance across all aspects, achieving a 100% success rate in assessment following successful standard retrieval. The other models also performed admirably in matching standards with data, maintaining overall success rates exceeding 95%. As illustrated in Figure 7, all models consistently showed slightly higher success rates in the “assessment” task compared to the “retrieval and comparison” task. This pattern can be attributed to the dependent relationship between assessment and retrieval–comparison processes; when the knowledge base is correctly retrieved, assessment results typically show minimal deviation. These two tasks are fundamentally complementary, and the full potential of LLMs can only be realized when both tasks achieve high levels of accuracy and efficiency.

### 4.3. Preliminary Implementation of Embodied Intelligence

**Figure 8:**
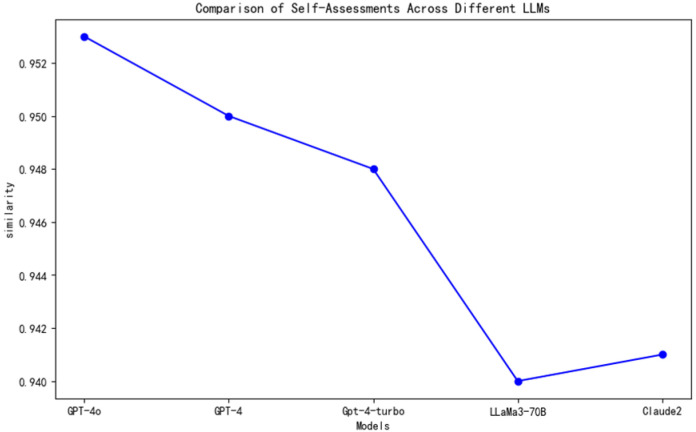
Line chart comparing self-assessments of different LLMs.

In the preliminary implementation of embodied intelligence, we adopted a self-assessment method based on LLMs to initially evaluate the degree of realization of embodied intelligence. This self-assessment method provides an effective metric for the realization of embodied intelligence. The core of the method lies in leveraging the LLMs’ ability to understand the content of the knowledge base and the accuracy of generating corresponding judgments. The line graph in Figure 8 shows that GPT-4o has a stronger capability to comprehend the content of the knowledge base and generate aligned judgments, reaching a high of 0.952, followed by GPT-4 at 0.950. GPT-4-turbo, LLaMa3-70B, and Claude2 also demonstrate commendable abilities. The high scores achieved by the models indicate that, with the advancement of artificial intelligence technology, LLMs are continuously improving in their ability to handle complex cognitive tasks.

**Figure 9:**
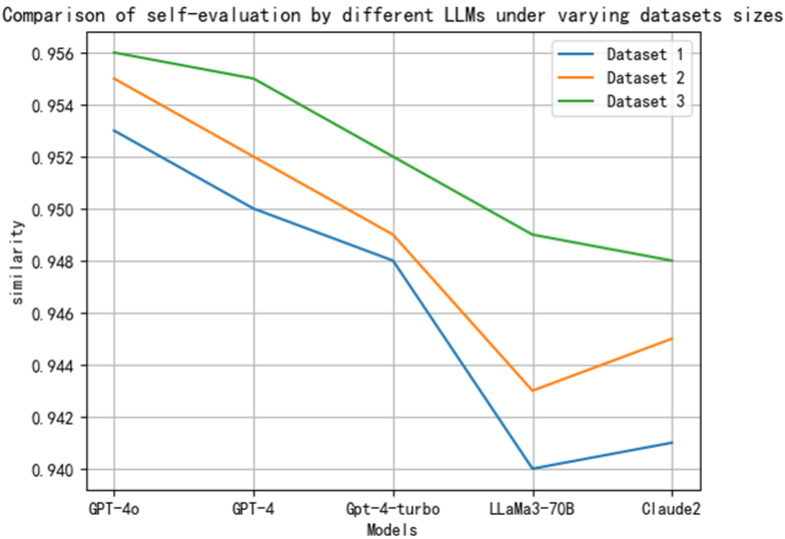
Self-assessments of different models under various datasets.

In the validation process, we employed a hierarchical dataset validation approach, dividing the datasets into three different scales for experimentation. The following is a detailed description of each group of datasets and an in-depth analysis of the model evaluation results:

The first dataset group: This dataset comprises 5000 data points from different measurement sites and types. This scale of dataset provides a fundamental research sample, allowing us to conduct an initial assessment of the model’s performance. As illustrated in Figure 9, at this stage, the model’s evaluation similarity reached a certain level, indicating that the model has a degree of effectiveness in handling smaller-scale data.

The second dataset group: Building upon the first dataset group, we increased the amount of data to 10,000 data points from different measurement sites and types. With the increase in data volume, the model’s evaluation similarity improved. This suggests that with the support of more data, the model is better able to learn and adapt to sensor data and environmental changes. At this stage, the interaction between the system and sensor data, as well as the environment, is more comprehensive, leading to a continuous increase in the similarity of the model’s self-assessment.

The third dataset group: We further expanded the data volume to 20,000 data points from different measurement sites and types. On this scale of dataset, the model’s evaluation similarity reached its highest level. This indicates that as the dataset grows larger, the model’s learning capability is fully utilized, enabling it to capture data features more accurately and thus improve the similarity of the evaluation results. Throughout this process, the system continuously interacts with sensor data and the environment, leading to an increasingly higher similarity in the model’s self-assessment, validating the model’s stability and reliability on large-scale datasets.

In summary, by comparing the validation results across datasets of varying scales (as shown in Figure 9), we can derive the following insights: as the dataset size increases, there is a marked improvement in the model’s performance. This is particularly evident when processing large-scale data, where the model demonstrates enhanced adaptability to complex environments and improves the similarity of evaluation outcomes. These findings provide a robust foundation for further model optimization and serve as a valuable reference for data processing in real-world application scenarios.

## 5. Evaluation

### 5.1. Evaluation Settings

To assess the accuracy and reliability of the embodied intelligent system’s judgment, we employed BLEU scoring, ROUGE evaluation, and Levenshtein distance metrics for evaluation.

The BLEU evaluation method measures translation quality by comparing the n-gram similarity between the machine-generated output and the human reference content. BLEU scores range from 0 to 1, with higher scores reflecting greater accuracy. An n-gram is a widely used model in natural language processing that extracts continuous sequences of characters or words from text. In BLEU evaluation, n-gram similarity is employed to compare the reference content with the machine-generated output. Specifically, the reference and output content are segmented into various n-grams, and their similarity is calculated. To prevent a score of zero when n increases, a smoothing function is applied for cases where n exceeds 4.

The calculation formula for BLEU is as follows:

(1) $$BLEU=BP\cdot \left(\sum_{n=1}^{N}w_{n}logp_{n}\right)$$

where BP is the length penalty term, known as the Brevity Penalty, defined as follows:

(2) $$BP=\left\{\begin{array}{c} 1,\;\;\;\;\;\;\;\;\;\;\;\;\;\;\;\;\;\;\;if\;c>r\\ exp\left(1-\frac{r}{c}\right),\;\;\;if\;c\leq r \end{array}\right.$$

where c is the length of the generated text, and r is the length of the reference text.

(3) $$p_{n}=\frac{\sum_{I}^{E}\sum_{k}^{K}min\left(h_{k}\left(c_{i}\right),\underset{k\in M}{\min}h_{k}\left(s_{i};j\right)\right)}{\sum_{i}^{E}\sum_{k}^{K}min\left(h_{k}\left(c_{i}\right)\right)}$$

Herein, E represents the total number of candidate texts, and K represents the total number of word groups; $h_{k}\left(c_{i}\right)$ indicates the frequency at which the k-th word group appears in the selected text $c_{i}$; $s_{i}$ represents a reference answer, where j∈M in M, and M denotes the number of reference answers; ${andh}_{k}\left(s_{i};j\right)$ represents the standard answer, indicating the frequency of occurrence of the k-th group of words in $\left(s_{i};j\right)$. In Equation (1), $w_{n}$ is the weight, usually using equal weights for each n-gram, i.e., $w_{n}=\frac{1}{n}$, where N is the order of the maximum n-gram (e.g., N = 4 means from 1 − g to 4 − g).

The ROUGE evaluation method was designed to emulate how human evaluators assess summary quality by measuring the overlap between system-generated content and reference content. The core metrics of ROUGE evaluation comprise recall, precision, and F1 score. ROUGE-N evaluates the N-gram overlap between system-generated content and reference content. This includes ROUGE-1 for unigram overlap, ROUGE-2 for bigram overlap, and ROUGE-L for longest common subsequence overlap.

The calculation formula for ROUGE-N is as follows:

(4) $$ROUGE-N=\frac{\sum_{S\in \left\{Reference\left.Summaries\right\}\right.}\sum_{{gram}_{n}\in S}{Cout}_{match\left({gram}_{n}\right)}}{\sum_{S\in \left\{Reference\left.Summaries\right\}\right.}\sum_{{gram}_{n}}Count\left({gram}_{n}\right)}$$

where the denominator is the number of n-grams, and the numerator is the number of n-grams that are common to both the reference content and the automatically generated content.

The calculation formula for ROUGE-L is as follows:

(5) $$\begin{array}{c} R_{lcs}=\frac{LCS\left(X,Y\right)}{m}\\ \;P_{lcs}=\frac{LCS\left(X,Y\right)}{n}\\ F_{lcs}=\frac{\left(1+\beta^{2}\right)R_{lcs}P_{lcs}}{R_{lcs}+\beta^{2}P_{lcs}} \end{array}$$

where $LCS\left(X,Y\right)$ represents the length of the longest common subsequence between X and Y, taking order into account. m,n represent the lengths of the reference content and automatically generated content, respectively (usually the number of words contained). $R_{lcs}$ and $P_{lcs}$ represent recall and precision, respectively. The final $F_{lcs}$ is what we call Rouge-L.

The Levenshtein distance, alternatively referred to as edit distance, represents a precise metric for quantifying the dissimilarity between two sequences (predominantly strings). This metric functions by computing the minimal number of single-character edit operations necessary to transform one string into another, encompassing operations such as insertion, deletion, and substitution of characters. Consequently, the Levenshtein distance not only quantifies character-level differences between strings, but also reflects their structural and content-based similarities to a significant degree. A lower Levenshtein distance typically indicates greater similarity between two sequences. This metric has emerged as an essential tool in various domains, particularly in natural language processing and error correction applications.

The formula for Levenshtein distance is as follows:

(6) $${lev}_{a,b}\left(i,j\right)=\left\{\begin{array}{c} \max\left(i,j\right)\;\;\;\;\;\;\;\;\;\;\;\;\;\;\;\;\;\;\;\;\;\;\;\mathrm{if}\;\min\left(i,j\right)=0,\\ \min\left\{\begin{array}{c} {lev}_{a,b}\left(i-1,j\right)+1\\ {lev}_{a,b}\left(i,j-1\right)+1\\ {lev}_{a,b}{(i-1,j-1)+1}_{a_{i}\neq b_{j}} \end{array}otherwise\right. \end{array}\right.$$

where: when $a_{i}=b_{j}$, $l_{\left(a_{i}\neq b_{j}\right)}$ is 0, otherwise it is 1, ${lev}_{a,b}\left(i,j\right)$ is the edit distance between the i-th character of a and the first j characters of b.

### 5.2. Evaluation Results

In this validation of the results, the dataset was set to include 22,000 data entries, with gas monitoring comprising 10,000 entries, including 4000 related to methane alarms, 3000 related to carbon monoxide alarms, and 3000 related to carbon dioxide alarms. Environmental monitoring consisted of 10,000 entries, with 5000 related to environmental temperature alarms, 5000 related to ventilation alarms, and 2000 normal data entries. The following are the evaluation results for each indicator.

**Figure 10:**
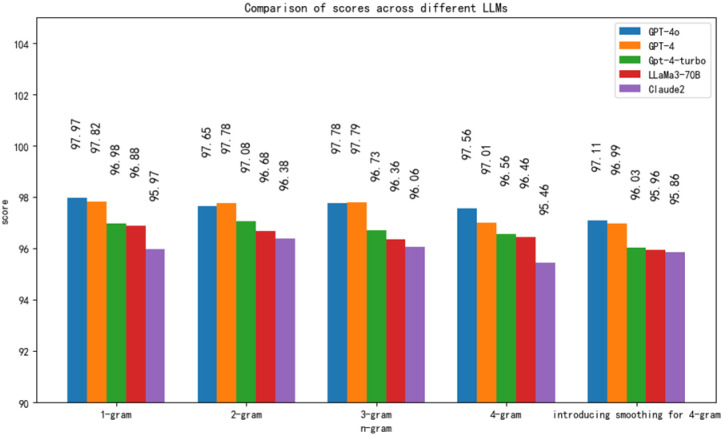
Scores of different LLMs on various grams of BLEU.

The BLEU evaluation method has been widely used across various tasks, such as summarization and text translation. Notably, it achieved a score of 0.88 in evaluating translations generated by ChatGPT-4 [47,48]. As shown in Figure 10 of this paper, the BLEU scores of the GPT-4o model are generally higher than those of the GPT-4 model across all n-gram levels, indicating that the GPT-4o model exhibits better performance in terms of coherence and accuracy. At the 1-g level, the GPT-4o model scores slightly higher than the GPT-4 model, with a small difference. The success rates of GPT-4-turbo, LLaMa3-70B, and Claude2 decrease in sequence, but overall, they remain quite satisfactory. This suggests that all models show similar performances in the selection of individual words. At the 2-g level, the GPT-4o model’s score once again slightly exceeds the GPT-4 model, indicating a slight advantage of GPT-4o in generating more natural phrase or word combinations, while GPT-4-turbo, LLaMa3-70B, and Claude2 do not perform as well as GPT-4, but still meet the requirements. At the 3-g level, the GPT-4o model’s score is significantly higher than that of the GPT-4 model, demonstrating its superiority in constructing longer and more complex sentence structures. At the 4-g level, although the GPT-4o model’s raw BLEU score is lower than that of the GPT-4 model, after the introduction of a smoothing function, its score surpasses that of the GPT-4 model. The smoothing function avoids the situation where n-grams receive no score. In conclusion, the GPT-4o model shows better performance in most cases, particularly in its ability to construct complex sentences.

From the comprehensive evaluation results of the ROUGE metrics in Table 2, we can observe that the GPT-4o model demonstrates slight advantages across various indicators. Specifically, in the ROUGE-1 assessment, both GPT-4 and GPT-4o models exhibit exceptional performance, achieving high standards with precision, recall, and F-scores exceeding 0.975, indicating superior text generation quality and coverage of the original text. While GPT-4-turbo, LLaMa3-70B, and Claude2 perform below GPT-4o and GPT-4, they maintain overall scores above 0.970. In the ROUGE-2 evaluation, despite more stringent criteria, the GPT-4 and GPT-4o models maintain high scores above 0.975, demonstrating both models’ effectiveness in capturing phrase-level semantic information, with GPT-4-turbo, LLaMa3-70B, and Claude2 following closely behind. Regarding the ROUGE-L metric, which measures the degree of matching between the longest common subsequence of model-generated text and reference text, GPT-4 and GPT-4o models achieve precision, recall, and F-scores no lower than 0.974. These results further validate their outstanding performance in terms of overall structural consistency. Although GPT-4-turbo, LLaMa3-70B, and Claude2 do not match the excellence of GPT-4o and GPT-4, they still achieve scores of 0.970.

Overall, the performance of models in the ROUGE evaluation is exceptional, with a very high degree of accuracy. This indicates that when LLM retrieves stored standards or records, the retrieved content is precise, and the judgments generated based on the content are also accurate.

As shown in Table 3, the Levenshtein distances for the models GPT-4o, GPT-4, GPT-4-turbo, LLaMa3-70B, and Claude2 are overall quite small, with the largest distance being only 1.094. This indicates that the judgments generated by the LLM’s analysis of the retrieved content are nearly identical to the original standard documents. This result further demonstrates that LLMs not only are capable of precisely understanding and interpreting input content, but can also make judgments with high accuracy to fulfill their functions. Among these, GPT-4o has the smallest Levenshtein distance, with a value of only 0.966.

## 6. Discussion

During the path-planning phase, the system demonstrated certain advantages. It is capable of dynamically adjusting the decision-making direction based on the current level’s demands and data changes, thereby achieving efficient path selection. However, as the hierarchy deepens and the tasks become more complex, the system experienced deviations in planning paths, resulting in a decrease in the success rate, but overall, the outcomes are still ideal.

In the standard and data-matching phase, different LLMs exhibit varying performance. The GPT-4o model has a higher success rate in both the retrieval and comparison phase and the evaluation and judgment phase than other models. This may be due to the GPT-4o model’s stronger capabilities in natural language understanding and reasoning.

Based on LLMs’ cognitive and natural language-processing capabilities, the system effectively manages path-planning tasks, while its logical reasoning abilities provide a foundation for data assessment. When employing different LLMs, the system demonstrates nearly identical success rates in planning-level path planning. However, at the action level, when executing specific assessment tasks, the evaluation metrics vary among models. During complex tasks, the system’s performance appears less exceptional compared to its execution of individual simple tasks. This phenomenon may be attributed to the accumulation of minor errors in each step of the complex replication process, ultimately affecting the quality of task completion. Nevertheless, from a holistic perspective, the effectiveness of the embodied intelligence assessment system remains commendable. While the system may exhibit certain limitations in handling complex tasks compared to single-task execution, its overall performance remains satisfactory. In general, GPT-4o demonstrates the most outstanding performance in the system, ranking first, followed by other models. Although different large models exhibit varying performance levels, they all maintain generally favorable results. This indicates that the system retains significant practical value and potential in addressing various tasks.

## 7. Conclusions

In the in-depth exploration of this paper, we focus on the application of embodied intelligence within multi-level architectural LLMs, particularly emphasizing its practical capabilities in intelligent assessment domains. We elaborate on the system’s methodology for efficient processing and precise evaluation of sensor-acquired data. This research conducts an in-depth assessment analysis of data from ten representative scenarios discussed in the paper. These analytical findings enable effective responses to various potential risks and challenges.

The design concept of the multi-level structure effectively enhances the system’s processing speed, accuracy, and stability, and achieves streamlined processing and specialized judgment. The powerful natural language understanding and reasoning capabilities of LLM provide the system with robust cognitive abilities, enabling it to effectively process multi-source sensor data and perform intelligent judgments. The combination of vector databases and relational databases, along with the application of RAG technology, greatly enhances the precision and applicability of the evaluation criteria and promotes the system’s utilization of historical experience. The human–machine interaction feedback mechanism ensures the system’s flexibility and adaptability and fosters continuous learning and optimization. The introduction of embodied intelligence allows the system to enhance its perception of environmental changes through physical interaction with the environment, and to realize more flexible and adaptive decision making.

The system demonstrates effective capabilities in identifying alarm data, exhibiting optimal performance across path-planning, knowledge base retrieval, and assessment processes. Its ability to assess data promptly and accurately reduces the risks associated with mine safety management. Through environmental interaction, the system efficiently learns sensor data characteristics and, through sustained interactive learning, develops the ability to reason about anomalous data patterns within specific time periods. For instance, the system has successfully learned to recognize ventilation variations during shift changes, identifying the three daily shift transition points. The system exhibits exceptional adaptability to environmental conditions and reliability in intelligent decision making. With continuous technological advancements and optimization, the capabilities of embodied intelligence in environmental perception, decision making, and autonomous learning will be further enhanced, paving the way for more intelligent, efficient applications and maintenance management processes. Future research will focus on integrating multi-level structures with large language models and exploring the potential applications of embodied intelligence across various domains.

Although the system demonstrates significant potential in the field of mine safety, we have also noted some limitations:

(1) Knowledge Base Limitations: The system’s accuracy and reliability are highly dependent on the quality of its knowledge base. Inaccurate or outdated information within the knowledge base may lead to imprecise assessment results. Given the rapid evolution of mining safety knowledge, regular updates to the knowledge base are essential to maintain its timeliness and accuracy.
(2) Model Limitations: LLM still exhibit constraints in their comprehension capabilities, potentially misinterpreting complex scenarios or ambiguous information, which may result in assessment errors. Additionally, the model’s reasoning capabilities are bounded by its training data, potentially limiting its effectiveness in addressing scenarios beyond its training scope.

To address these limitations, several improvement measures can be implemented: establishing and refining data collection and management mechanisms to ensure high data quality; conducting regular knowledge base updates to maintain its currency; researching and developing more advanced language models to enhance their comprehension and reasoning capabilities; and strengthening model interpretability to boost user confidence in system outcomes. Through these continuous enhancement efforts, the system shows promise in playing an increasingly crucial role in mine safety, providing more robust technical assurance for safe mining operations.

## 8. Future Outlook

The Embodied Intelligence System for Coal Mine Safety Assessment Based on Multi-Level LLMs proposed in this study demonstrates significant potential in the field of mine safety. However, this is merely the beginning, with numerous directions worthy of exploration and expansion in the future.

Firstly, the improvement and updating of the knowledge base will be key to the system’s sustained development. As knowledge in the field of mine safety continues to be updated and advanced, the system will need to regularly update its knowledge base to maintain its relevance and accuracy. The scale and content of the knowledge base will need to be continuously expanded to cover a wider range of scenarios and issues. Secondly, the optimization of human–machine interaction will enhance the system’s ease of use and adaptability. The application scenarios of the system can be further expanded. The design philosophy and technical methods of this system can be applied to other fields, such as other types of safety monitoring, equipment fault diagnosis, environmental monitoring, etc. This will enable the system to provide safety assurance for more industries and fields.

The integration of embodied intelligence will substantially enhance the system’s perceptual and decision-making capabilities. The embodied intelligence technologies can be iteratively optimized through advanced approaches such as multi-modal perception and reinforcement learning algorithms, enabling the system to achieve more sophisticated environmental awareness and facilitate more precise decision-making processes.

## Figures and Tables

**Table 1:** Classification of monitoring data for 10 scenarios.

Gas Monitoring	Environmental Monitoring	Structural Monitoring	Water Body Monitoring	Pressure Monitoring
Ambient Methane, High and Low Concentration Methane, Laser-Detected Methane, CO, H_2_S, O_2,_ CO_2,_ Pipeline Methane, Pipeline Carbon Monoxide, H_2,_ NO_2,_ SO_2,_ He, N_2,_ C_2_H_4,_ C_2_H_6_	Wind Velocity, Ambient Temperature, Humidity, Air Volume/Volumetric Airflow, Wind Direction, Precipitation, Smoke Concentration/ Particulate Matter	Roof Strata Separation Displacement, Dam Structure Displacement	Pool Water Level, Aquatic Temperature, Reservoir Storage Level, Saturation Line, Aqueous Quality Indices, Discharge Flow Rate, Volumetric Flow Rate	Subatmospheric Pressure, Pipeline Internal Pressure, Hydraulic System Pressure, Pressure Magnitude
**Level Monitoring**	**Equipment Status** **Monitoring**	**Stress** **Monitoring**	**Flow Monitoring**	**Other Monitoring** **Parameters**
Coal Seam Elevation, Liquid Surface Elevation, Material Spatial Positioning	Voltage, Frequency, Electrical Current, Localized Ventilation Unit, Ventilation Door, Air Duct Status, Equipment Operational State, Main Ventilation Fan, Power Feeder, Audible-Visual Alarm, Substations, Electrical Power System State	Surrounding Rock Stress, Borehole Wall Stress State, Rock Bolt Axial Stress	Composite Methane Gas Flow Rate, Pure Methane Gas Flow Rate, Pipeline Volumetric Flow Rate, Volumetric Flow Rate	Noise, Motor Temperature, Coal Storage Utilization Ratio, Aperture Degree, Height, Production Volume, Gas Extraction Volume

**Table 2:** Scores of different LLMs on ROUGE.

LLM	Indicator	ROUGE-1	ROUGE-2	ROUGE-L
GPT-4o	precision	0.979	0.977	0.977
	recall	0.979	0.975	0.976
	F-measure	0.979	0.976	0.977
GPT-4	precision	0.978	0.975	0.974
	recall	0.977	0.976	0.975
	F-measure	0.977	0.975	0.976
gpt-4-turbo	precision	0.973	0.974	0.973
	recall	0.975	0.975	0.973
	F-measure	0.972	0.973	0.971
LLaMa3-70B	precision	0.971	0.974	0.972
	recall	0.972	0.972	0.971
	F-measure	0.970	0.971	0.970
Claude2	precision	0.969	0.970	0.970
	recall	0.968	0.971	0.971
	F-measure	0.970	0.973	0.972

**Table 3:** Scores of different LLMs on Levenshtein distance.

LLM	Levenshtein Distance
GPT-4o	0.966
GPT-4	1.008
Gpt-4-turbo	1.076
LLaMa3-70B	1.086
Claude2	1.094

## Data Availability

The data and code are available at the following address: https://gitee.com/sun-yi-laboratory-team/assessment_-jfx.git (accessed on 18 December 2024).
